# Machine learning for optimized individual survival prediction in resectable upper gastrointestinal cancer

**DOI:** 10.1007/s00432-022-04063-5

**Published:** 2022-05-26

**Authors:** Jin-On Jung, Nerma Crnovrsanin, Naita Maren Wirsik, Henrik Nienhüser, Leila Peters, Felix Popp, André Schulze, Martin Wagner, Beat Peter Müller-Stich, Markus Wolfgang Büchler, Thomas Schmidt

**Affiliations:** 1 Department of General, Visceral and Transplantation Surgery, University Hospital of Heidelberg, Im Neuenheimer Feld 420, 69120 Heidelberg, Germany; 2 Present Address: Department of General, Visceral and Cancer Surgery, University Hospital of Cologne, Kerpener Straße 62, 50937 Cologne, Germany

**Keywords:** Gastric cancer, Esophageal cancer, Machine learning, Survival analysis, Oncological outcome

## Abstract

**Purpose:**

Surgical oncologists are frequently confronted with the question of expected long-term prognosis. The aim of this study was to apply machine learning algorithms to optimize survival prediction after oncological resection of gastroesophageal cancers.

**Methods:**

Eligible patients underwent oncological resection of gastric or distal esophageal cancer between 2001 and 2020 at Heidelberg University Hospital, Department of General Surgery. Machine learning methods such as multi-task logistic regression and survival forests were compared with usual algorithms to establish an individual estimation.

**Results:**

The study included 117 variables with a total of 1360 patients. The overall missingness was 1.3%. Out of eight machine learning algorithms, the random survival forest (RSF) performed best with a concordance index of 0.736 and an integrated Brier score of 0.166. The RSF demonstrated a mean area under the curve (AUC) of 0.814 over a time period of 10 years after diagnosis. The most important long-term outcome predictor was lymph node ratio with a mean AUC of 0.730. A numeric risk score was calculated by the RSF for each patient and three risk groups were defined accordingly. Median survival time was 18.8 months in the high-risk group, 44.6 months in the medium-risk group and above 10 years in the low-risk group.

**Conclusion:**

The results of this study suggest that RSF is most appropriate to accurately answer the question of long-term prognosis. Furthermore, we could establish a compact risk score model with 20 input parameters and thus provide a clinical tool to improve prediction of oncological outcome after upper gastrointestinal surgery.

**Supplementary Information:**

The online version contains supplementary material available at 10.1007/s00432-022-04063-5.

## Introduction

The demand for adequate prediction of oncological outcome after upper gastrointestinal surgery will increase due to a rising global incidence of esophageal and gastric carcinomas (Malhotra et al. 2017). In this regard, patient profiles are heterogenous due to varying in-hospital courses and additional information about clinical and histopathological characteristics, which make the individual prognosis difficult to predict. While most patient characteristics are hardly improvable and are rather predetermined by the nature of the disease, there might be critical periods during the treatment of a malignancy such as the phase of oncological resection when many fundamental conditions for long-term outcome are set. This way, the in-hospital stay for surgery is literally an incisive event in the clinical course of one individual patient. However, surgical morbidity can significantly influence the overall and long-term survival in gastric cancer (Kulig et al. 2021). Eventually, clinicians are confronted with a patient curious to know what the information gathered so far from surgery actually means for his or her individual prognosis. While the patient is then frequently referred to the oncologist who will further supervise the prospective course, there may be a more concrete estimation to offer at the time of discharge. Surgeons with an oncologic focus need the tools to provide these answers to adequately care for their patients.

The motivation to accurately predict the individual prognosis of one patient also derives from the growing interest to assess individualized patient profiles in times of personalized medicine (Hu and Steingrimsson 2018). Due to the complexity of each patient case, differences in individual prognosis could eventually implicate different (adjuvant) therapeutic regimens. In this context, the vast amount of clinical data should be viewed as another component of “omics”-based technology and as another step toward personalized treatment.

Machine learning techniques can help to determine significant associations based on complex and vast data (Kourou et al. 2015). Retrospective machine learning studies have already delivered suggestive data that artificial intelligence may be promising in case of multiple potential factors with unclear relations to each other. Since the proposal of the proportional hazards model by David Cox (1972) there were many advances to improve survival prediction, especially since computational possibilities have increased. Among those, machine learning algorithms have recently shown promising results (Zhu et al. 2020). According to the current literature there are many works regarding the automated prediction of time-dependent, censored data with machine learning methods. Besides survival being the most typical and classic scenario, there have also been approaches to predict heart failure (Panahiazar et al. 2015), kidney transplant durability (Sekercioglu et al. 2021) and many other settings. Spooner et al. were able to demonstrate that machine learning algorithms for survival analysis were applicable to the clinical task of predicting dementia (Spooner et al. 2020). Based on two separate datasets, it was possible to reach a concordance index (*c*-index) of 0.82 and 0.93, respectively.

However, the prediction of oncological outcome after diagnosis of a malignant disease is still the most common target variable in machine learning studies. To this date, there are only few medical publications in the current literature dealing with machine learning models for survival analysis of oncologically resected upper gastrointestinal cancers. Akcay et al. have analyzed gastric cancer patients after chemoradiation with a comparable methodology (Akcay et al. 2020) and tested a random forest algorithm to predict tumor relapse in terms of distant metastases or peritoneal recurrence. Compared to other algorithms such as logistic regression, multilayer perceptron and extreme gradient boosting the random forest scored highest with an area under the curve (AUC) of 0.97 for peritoneal recurrence. However, the random forest algorithm did not show convincing results for overall survival with an AUC of only 0.59 and the authors did not apply a random survival forest methodology. Jiang et al. have utilized a LASSO cox analysis to predict oncological outcome of gastric cancer patients analyzing the radiomic signature of PET computer tomography (Jiang et al. 2018). It is notable that the authors evaluated imaging data and reached a powerful prediction for disease-free survival and overall survival with a *c*-index of 0.786. In the current literature, feature selection and extraction methods both perform poorly on low case numbers such as 500 or less individuals (Pölsterl et al. 2016). Above this minimum amount of 500 cases, machine learning methods should probably yield similar results and the final strategy to be chosen should rather depend on the individual data, the optimization process and the reached performances of the models.

To our knowledge, the topic of survival prediction in case of oncologically resected upper gastrointestinal cancer is not sufficiently covered by machine learning methods. However, the opportunity of pre- and postoperatively supervising an individual patient course offers an ideal setting to predict long-term outcome based on the collected data. The aim of this study was therefore to apply various machine learning algorithms to optimize survival prediction after oncological resection of gastroesophageal cancer compared to previously established methods.

## Material and methods

### Data collection and follow-up

Eligible subjects of this study were patients with distal esophageal, gastroesophageal junction or gastric cancer who underwent oncological resection between September 2001 and December 2020 at Heidelberg University Hospital, Department of General Surgery. All patients provided written consent for data collection and analysis. The data was initially collected prospectively in a clinical database. The trial protocol was approved by the ethics committee at the University of Heidelberg (committee’s approval: S-635/2013) and was performed in accordance with the Declaration of Helsinki, Good Clinical Practices as well as local ethics and legal requirements. To acquire long-term survival data, all patients were systematically followed up via continuous surveys. The relevant survival data for this patient collective was gathered until October 2020.

### Inclusion and exclusion criteria

Out of the main collective, only those patients were further taken into account with a preoperatively diagnosed and histologically proven adenocarcinoma of the distal esophagus, the esophagogastric junction or stomach. Patients were excluded who had squamous cell carcinoma at any location. Also, only radical oncological resections were evaluated as opposed to exploratory laparotomies with palliative treatment. Furthermore, the type of oncological resection was limited to (sub-) total gastrectomy, gastrectomy with transhiatal extension, proximal gastrectomy, abdominothoracic esophagectomy and discontinuity resection of the esophagus. Variables with a missingness greater than 50% were generally excluded from further analysis. Also, patients were excluded with a case-specific missingness of more than 10%.

### Data structure

Eventually, the dataset had a total of 117 features and 1360 patients. Out of the whole dataset, 92 variables were categorical (with 77 Boolean variables) and 25 variables were numeric. Table 1 gives a short overview of the parameters including all dependent variables (or end points, respectively). There were obvious survival predictors and other variables which had to be excluded due to collinearity (see Supplementary Fig. 1). Regarding survival data, 53.5% of the records were censored with an equal distribution over the whole time span. Supplementary Fig. 2 demonstrates the censored data added on top of the patients that verifiably passed away and plotted against the follow-up period.

**Table 1:** Overview of independent and outcome variables

Biometric variables
Height, weight, body mass index (BMI), age, sex
Preoperative variables
Past medical history (cardiovascular, pulmonary, metabolic and renal preconditions), tumor diagnosis, preceding malignant disease, cTNM classification, histology (Laurén type, signet cell component), neoadjuvant therapy (components, radiotherapy, completeness)
(Intra-)operative variables
Time between diagnosis and resection, type of operation, extent of resection, anatomical reconstruction, duration of surgery, intraoperative complication, blood loss and transfusion
Postoperative variables
Days on ICU and ward, postoperative complications (according to Clavien–Dindo and additional 38 binarily classified types), pTNM classification, lymph node ratio (positive lymph nodes divided by resected), grading, R status, histology (Laurén type, signet cell component, tumor regression), post-discharge problems
Outcome variables
Vital status, overall survival, no evidence of disease, time until tumor relapse, 30-day mortality and in-hospital mortality

### Missingness and imputation

After the application of the above-mentioned criteria, there was a remaining missingness in 2,033 datapoints and thus an overall missingness of 1.3% without any duplicate values (see Supplementary Fig. 3). For imputation, missingness at random was assumed and multiple imputations with *n* = 1000 iterations via IterativeImputer from the Scikit learn package (Pedregosa et al. 2011) were performed on Python 3.9 (Rossum and Drake 2009).

### Statistical analysis

All statistical analyses were performed on Python 3.9 with packages such as scikit-learn 0.24.2 by Pedregosa et al. (2011). To compare various machine learning algorithms, the package PySurvival by Fotso et al. (2019) was implemented. The performances of the different algorithms were scored according to the concordance index (Uno et al. 2011) which can be regarded as the most important outcome parameter in this study. Hyperparameter optimization and cross-validation are all based on this concordance index, or *c*-index. The *c*-index is a generalization of the area under the receiver operating characteristic (ROC) curve (AUC) for censored outcome parameters such as right-censored survival data. It represents the global assessment of the model’s discrimination power which is the model’s ability to correctly provide a reliable ranking of the survival times based on the individual risk scores (Stephane Fotso 2019). The modified version of the *c*-index by Uno et al. is implemented in PySurvival and thus in this study and works as a simple nonparametric estimator free of censoring under the assumption of random censorship in general.

Furthermore, the inverse probability of censoring weights (IPCW *c*-index) was considered as an alternative to the *c*-index which is independent of the distribution of censored cases in the test data. This was relevant due to the comparably high prevalence of censored time points in the present dataset (see Supplementary Fig. 2). To evaluate another performance score, the integrated Brier-score (further abbreviated as IBS) was utilized (Steyerberg et al. 2010). The IBS was eventually plotted to demonstrate prediction error with a cut-off limit of 0.25 considered as critical. Furthermore, the actual survival function was plotted against the predicted function by the model. Finally, time-dependent evaluation of the area under the curve (AUC) was performed for selected machine learning ensembles provided by the scikit-survival package version 0.15.1 (Pölsterl 2020).

### Feature selection and importance

We did not perform feature selection before application of algorithms since previous works by Spooner et al. have shown that feature selection in this scenario does not significantly improve model performance (Spooner et al. 2020). The authors have applied seven different feature selection algorithms before running 5-fold cross-validation with 5 repeats. The results did not show any significant improvement of test statistics and most machine learning algorithms have a feature selection method internalized already. Moreover, we tested feature selection methods such as the Boruta variable selection algorithm (Kursa and Rudnicki 2010) for short-term outcome parameters such as in-hospital mortality and 30-day mortality. The results of the outcome analyses based on the selected variables are not shown in this manuscript because of unconvincing results. More importantly, these methods are not appropriate to deal with right-censored survival data and some of the presented machine learning methods have already internalized feature selection algorithms such as the different importance modes of the survival forests.

For eventual interpretation of feature importance, we identified the 20 most relevant predictors after fitting the corresponding machine learning algorithm via permutation-based importance evaluation provided by ELI5 adapted to the scikit-learn package (Arya et al. 2020). The algorithm computes feature importance for any black-box estimator by measuring how the score decreases when a feature is not available.

### Machine learning algorithms

The classic Cox proportional hazards model introduced by David Cox in 1972 is probably the most popular survival prediction model with an easy to interpret statistic (Cox 1972). We also tested a non-linear Cox proportional hazards model (also called DeepSurv) which is a multilayer perceptron (Katzman et al. 2018). However, the authors’ work is also known for the focus on recommender functions and treatment decision making. The linear multi-task logistic regression (L-MTLR) was introduced by Yu et al. in 2011 and operates on the basis of multiple logistic regressions (Yu et al. 2011). It may be considered as another general alternative to the Cox regression model. To increase flexibility, the neural multi-task logistic regression (N-MTLR) was introduced in 2019 (Stephane Fotso 2019). To fully represent all available survival prediction models, we also included one parametric model, the Gompertz model, as the relatively strongest test statistic compared to the Weibull and exponential model which are not demonstrated in this work. Last but not least, several random survival forest models were analyzed including the classic random survival forest (RSF) by Ishwaran et al. (2008). The Conditional Survival Forest model was developed by Wright et al. to improve splitting of the RSF by applying maximally selected rank statistics for the split point selection (Wright et al. 2017). The Extra Trees incorporated in PySurvival are an extension of the Extremely Randomized Trees (Geurts et al. 2006) which is a supervised learning method with almost totally randomized decision trees. The model is eventually independent of the output values of the learning sample and impresses with its high computational efficiency.

### Hyperparameters and optimization

All machine learning methods provided by PySurvival need specific hyperparameters to be optimized and defined before fitting the model. We performed hyperparameter optimization through defining the closest neighbor parameter values for each algorithm. Thereafter, the optimization was executed via Halving Grid Search included in the latest version of scikit-learn (Pedregosa et al. 2011). Halving Grid Search operates via successive halving of the candidate hyperparameters and their effect on the final test statistic as measured by the *c*-index. Although Halving Grid Search was not yet applied in many scientific works, it has already been shown to yield equivalent results for hyperparameter tuning compared to usual brute-force Grid Searching, however, with a much higher computation efficiency (Sraitih et al. 2021).

Some of the optimized hyperparameters will be presented in this paragraph. After hyperparameter optimization, the non-linear Cox proportional Hazards (DeepSurv), linear multi-task regression (LMTR), neural multi-task regression (NMTR) and parametric gompertz model used the so-called “Glorot” or “Xavier” uniform method for initialization (Glorot and Bengio 2010) which follows the Sigmoid activation function. The optimal initialization method for the standard Cox proportional hazards model was determined as “zeros” with initial setting of the tensors to 0. Other important hyperparameters for the above-mentioned five models were learning rate, number of epochs and last but not least L2 regularization to fight overfitting through minimization of weights. All survival forest models were fitted with an optimized number of trees of 200. The extra survival trees model (EST) used the “impurity corrected” method for variable importance which is a bias correction for the Gini variable in classification trees introduced by Sandri et al. (Sandri and Zuccolotto 2008). However, the optimal importance mode for conditional survival forest (CSF) and random survival forest (RSF) was determined to be “normalized permutation” which is a normalized version of the permutation importance originally introduced by Breiman et al. (Breiman 2001). While the three survival forest models used all available features, they differed in the determined minimum node sizes which were 15 for the CSF, 8 for the EST and five for the RSF. All hyperparameters are demonstrated in Supplementary Table 2.

## Results

The study included 1360 patients with oncological resection between 2001 and 2020 and 117 variables. Table 2 summarizes all patient characteristics with according percentage of missingess which were mainly evaluated as potentially relevant predictors for the machine learning models except for obvious outcome parameters.

**Table 2:** Overview of selected patient characteristics and percentage of missingness

*n* = 1360	Mean/median/frequency, (95% confidence interval)	Missingness (%)
Biometric variables		
Weight	77.7 kg (54.0–104.0)	0.0
Height	1.72 m (1.58–1.87)	1.0
Age	62.35 years (41.0–80.0)	0.0
Sex	Male (71.4%), female (28.6%)	0.0
Preoperative variables		
ASA score	1 (1.7%), 2 (47.9%), 3 (46.9%), 4 (2.3%)	1.2
Past medical history	See Supplementary Table 1	Max. 0.2
cT status	1 (7.1%), 2 (20.1%), 3 (57.2%), 4 (10.9%)	4.7
cN status	0 (35.7%), 1 (61.5%), + (0.4%)	2.4
cM status	0 (87.7%), 1 (11.9%)	0.4
Neoadjuvant therapy	Yes (55.0%), no (45.0%)	0.0
Chemotherapeutics	See Supplementary Table 1	Max. 2.1
Radiation	Yes (3.5%), no (96.2%)	0.4
(Intra-)operative variables		
Time diagnosis to resection	82.84 days (11.0–168.1)	0.1
One- vs. two-cavity surgery	One (71.1%), two (28.9%)	0.0
Operation type	Subtotal gastrectomy (21.5%) Total gastrectomy (26.4%) Transhiatal extended gastrectomy (21.8%) Ivor–Lewis esophagectomy (27.8%) Other types (2.6%)	0.0
(Locally) extended resection	Yes (28.5%), no (71.2%)	0.3
Intraoperative complications	Yes (7.8%), no (92.1%)	0.1
Duration of surgery	275.29 min (150.0–479.3)	0.9
Intraoperative blood loss	622.93 ml (100.0–1500.0)	19.2
Postoperative variables		
Duration of stay on ICU	6.89 days (0.0–29.0)	4.7
Duration of stay on ward	20.26 days (9.0–49.6)	0.7
Postoperative complications	See Supplementary Table 1	Max. 0.2
pT status	0/1 (22.3%), 2 (14.2%), 3 (47.3%), 4 (16.2%)	0.0
pN status	0 (42.1%), 1 (16.3%), 2 (15.0%), 3 (26.5%)	0.1
Lymph node ratio	18.25% (0.0–74.1)	0.1
pM status	0 (89.7%), 1 (10.3%)	0.1
R status	0 (81.5%), any 1/2/*X* (18.5%)	0.0
Outcome variables		
Status dead vs. alive/censored	Alive (51.5%), dead (46.1%)	2.4
Documented survival time	39.69 months (2.89–109.88)	1.0

*ASA* American Society of Anesthesiology, *ICU* intensive care unit

**Fig. 1:**
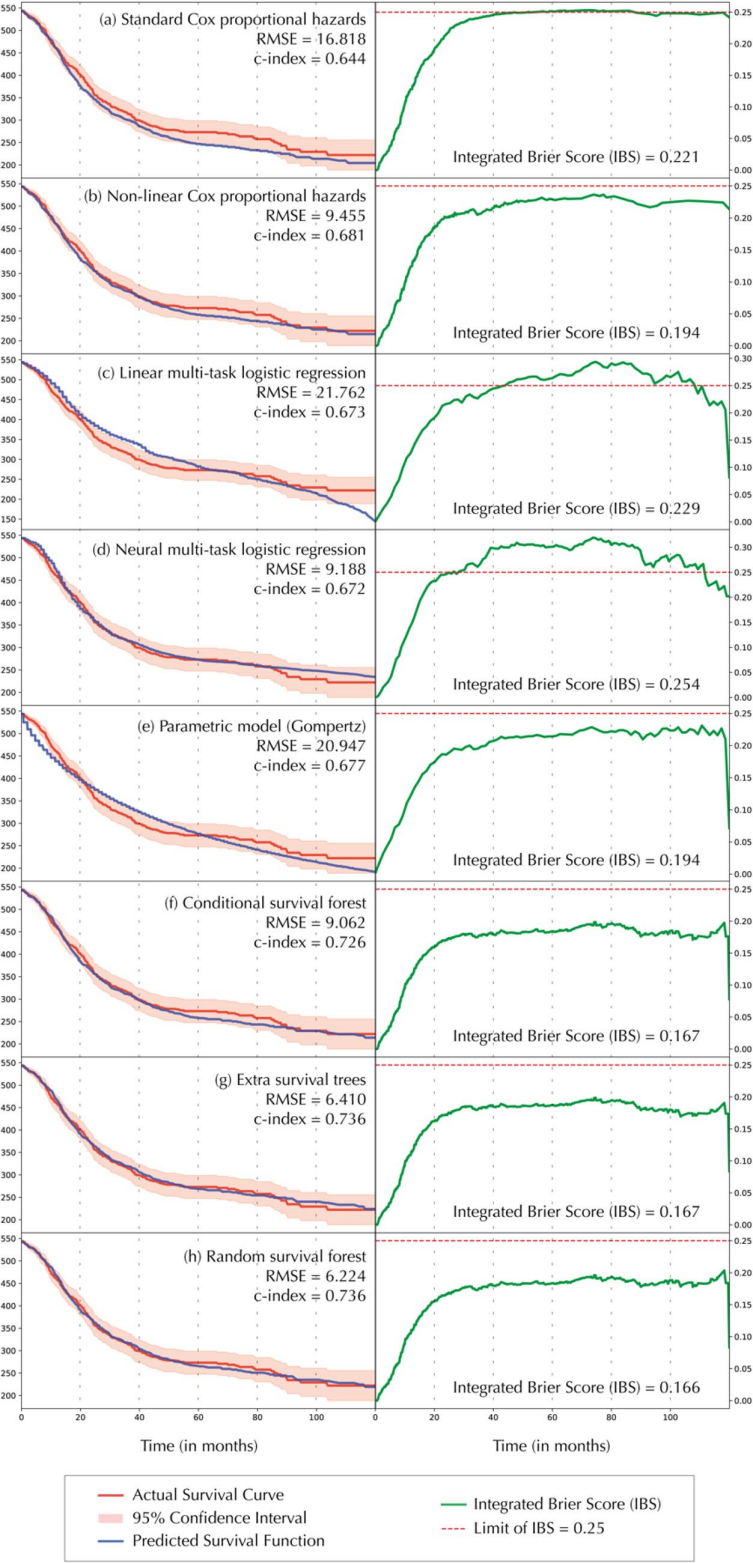
**a–h** Overview of machine learning algorithms and performances. *RMSE* root-mean-square error, *IBS* integrated Brier score

First of all, we tested the introduced machine learning algorithms which are demonstrated with the according test statistic in Fig. 1. The standard Cox proportional hazards (CPH) model is demonstrated in Fig. 1a and reached a *c*-index of 0.645 and an integrated Brier score (or IBS) of 0.221. The prediction error curve is depicted in the last column of Fig. 1 with the values for the Brier score as an integral. The non-linear Cox proportional hazards model (see Fig. 1b) was able to improve the statistic with a *c*-index of 0.681 and an IBS of 0.194. Note that while both Cox proportional hazards models initially have a comparably good score, the integral reaches the critical limit of 0.25 as the timespan reaches 10 years after cancer diagnosis.

Compared to the first two calculations, the *c*-index could not be improved by linear multi-task logistic regression (see Fig. 1c, *c*-index = 0.673, IBS = 0.229). However, the neural multi-task logistic regression reached a clearly better prediction of the actual survival function with a root-mean-square error (RMSE) of the actual predicted survival curve of 9.188 (see Fig. 1d, *c*-index = 0.672, IBS = 0.254). Note that both regression methods also exceed the IBS cut-off value of 0.25 which is generally considered as an acceptable limit. The Gompertz model as an example for a parametric model was not able to significantly improve the test statistic and showed relatively weak results (see Fig. 1e, *c*-index = 0.677, IBS = 0.194).

However, the CPH models could be outperformed by all three survival forest methods with the RSF being the strongest prediction model (see Fig. 1h, *c*-index = 0.736, IBS = 0.166). The Extra Survival Trees (see Fig. 1g, *c*-index = 0.736, IBS = 0.167) and the Conditional Survival Trees (see Fig. 1f, *c*-index = 0.726, IBS = 0.166) showed similarly strong results which could still not outperform the RSF even after hyperparameter optimization. The accurate prediction by RSF is again underlined by the direct comparison between actual and predicted survival function demonstrated in the second column. Here, the root-mean-square error (RMSE) of the RSF was calculated as 6.224.

To establish a more practical and compact approach, we selected the most important features identified by the RSF. Table 3 shows the permutation-based importance of the 20 most important parameters identified by the RSF model. While the individual weights of the predictors are not remarkably high (except for lymph node ratio), the RSF model can still build the prediction based on all variables of the dataset. The weights of the predictors are to be interpreted in a fashion that for instance the omittance of lymph node ratio in the model would evoke a change of the resulting *c*-index by 0.118 with the specified 95% confidence range.

**Table 3:** Most important 20 predictors based on permutation-based importance scoring for random survival forest

Predictive feature	Weight	Lower 95% CI	Upper 95% CI
Lymph node ratio (positive/total)	0.1183	0.1016	0.1350
Intraoperative blood loss	0.0060	−0.0009	0.0129
(y)pT4 status	0.0057	0.0002	0.0112
Age (at diagnosis)	0.0049	0.0035	0.0063
(y)pT3 status	0.0042	−0.0004	0.0088
Intraoperative peritoneal carcinosis	0.0040	0.0033	0.0047
Postoperative sepsis	0.0038	−0.0003	0.0079
cM+ status	0.0036	−0.0009	0.0081
Duration of in-hospital stay	0.0033	0.0028	0.0038
Duration of stay on ICU	0.0033	0.0004	0.0062
Major postoperative complications	0.0032	0.0020	0.0044
Any R+ status	0.0026	−0.0024	0.0076
Local R1 status	0.0021	0.0004	0.0038
(y)pM1 status	0.0020	0.0007	0.0033
(y)pT1 status	0.0014	0.0011	0.0017
Intraoperative blood transfusion	0.0013	0.0007	0.0019
Severe pre-existing diseases	0.0006	0.0002	0.0010
Siewert type I junction cancer	0.0006	−0.0004	0.0016
Cardiac postoperative adverse events	0.0005	0.0001	0.0009
ASA grade 3	0.0005	0.0000	0.0010

*ICU* intensive care unit, *ASA* American Society of Anesthesiology

**Fig. 2:**
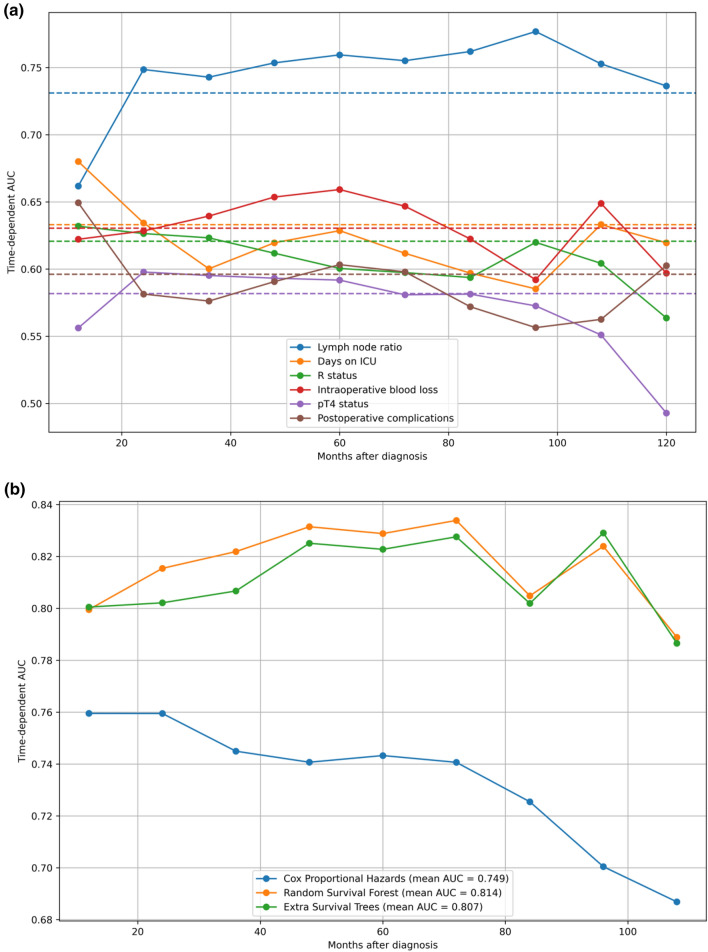
**a** Time-dependent AUC of the six most important variables according to permutation-based importange (see Table 3). **b** Time-dependent AUC of different models. *AUC* area under the curve; *ICU* intensive care unit

The time-dependent area under the curve (AUC) is separately demonstrated for the six most important predictors. It is remarkable that the AUC factors such as duration of intensive care, postoperative complications and intraoperative blood loss lose their predictive value rapidly as time passes. In contrast, the significance of lymph node ratio increases postoperatively and stays stable on an AUC level above 0.65 more than 5 years after cancer diagnosis (see Fig. 2a).

While predictors have a time-dependent importance, the prediction models also show a time-dependent accuracy. Of note, the RSF algorithm outperforms the CPH model on the time-dependent scale with an AUC of 0.821 (as opposed to 0.720 for the CPH model, see Fig. 2b). The remaining machine learning models also showed better time-dependent performances than CPH, but were not able to outperform the RSF algorithm. While all models were able to predict survival with a very high score above 0.9 in the very first months, the predictions generally tended to decrease in accuracy while time progressed. However, the long-term survival prediction was most successful according to the RSF model.

**Fig. 3:**
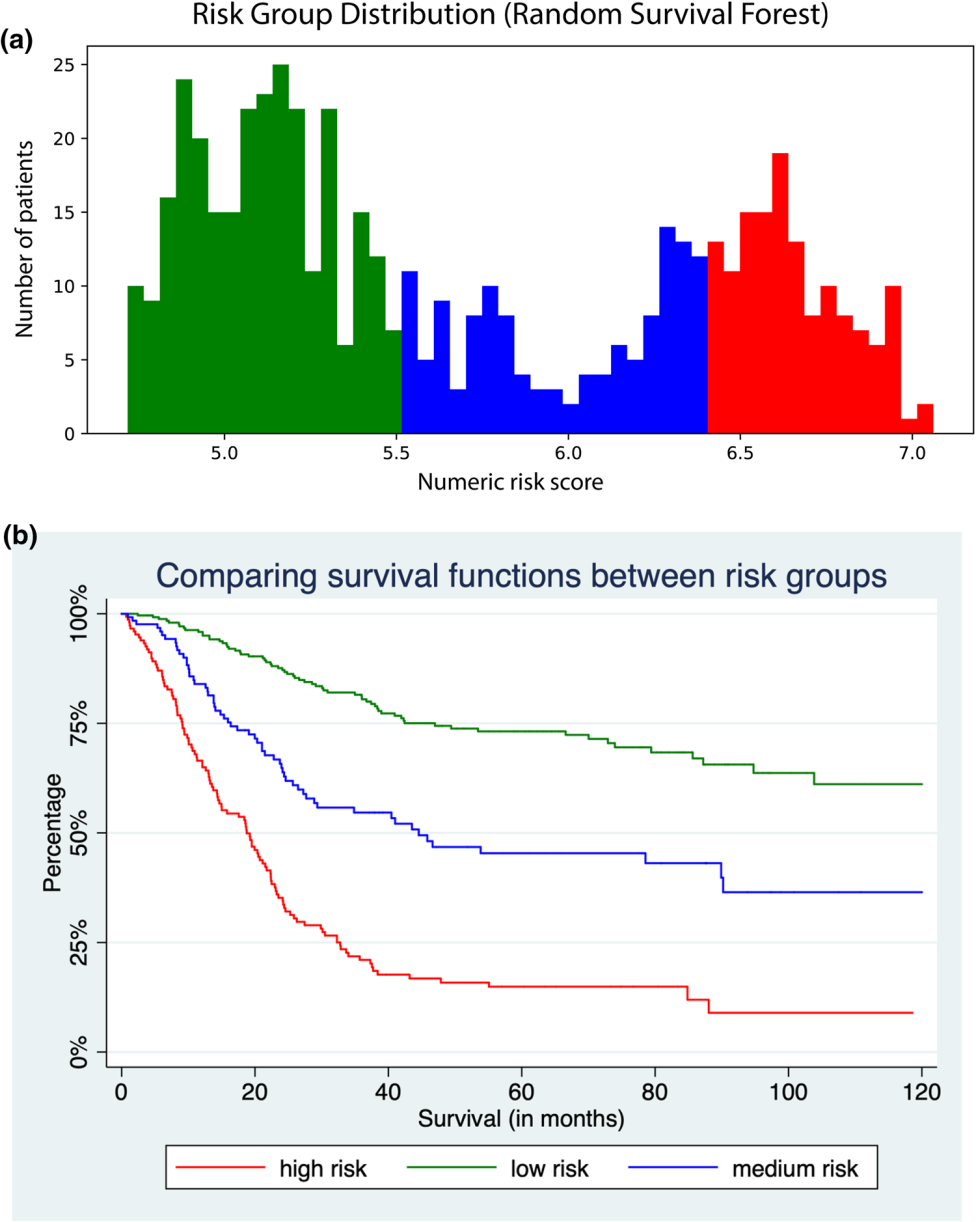
**a** Distribution of risk groups based on survival risk score from random survival forest utilizing all independent variables. **b** Differences in survival of the same risk score shown for the test group

Figure 3a shows a risk scoring model based on the test statistic calculated by the RSF algorithm. A numeric risk score is assigned to each patient ranging from 4.7 to 7.1. Three different colors were utilized to depict the low-, medium- and high-risk group. The differentiation of three groups was performed manually based on the distribution of risk scores. Figure 3b shows the survival curves of all individuals classified by the scoring system as low, medium or high-risk within the same predefined test group. The three survival curves differ significantly according to the log-rank test (*p* < 0.0001) with the low-risk group having a 5-year survival rate of 73.18%, the medium-group showing 45.39% and the high-risk group finally 14.87%. Median survival time was 18.754 months in the high-risk group, 44.557 months in the medium-risk group and incalculable in the low-risk group since the survival rate did not fall below 50% in the observed long-term time interval of 10 years. Also, the survival curves can be separated into more groups to enable a further stratification of risk groups (not shown).

Finally, the 20 most relevant predictors from the permutation-based importance scoring (see Table 3) were selected to establish a more compact RSF model. Supplementary Fig. 4 shows the distribution of the risk scores resulting from the compact RSF model. A risk score between 0 and 5.3 was considered as low-risk, a score between 5.3 and 6.1 as medium-risk and a score above 6.1 as high-risk for decease after resection. The according survival curves of the three different groups from the test cohort are demonstrated in Supplementary Fig. 5. The low-risk group had an incalculable median survival longer than 10 years, the medium-risk group had a median survival of 85.639 months and the high-risk group 20.721 months.

## Discussion

To improve the prediction of overall survival after gastroesophageal cancer resections we tested eight machine learning algorithms in this retrospective survival analysis to determine the best prediction model for oncological outcome. The Cox proportional hazards (CPH) model is by far the most frequently applied model to evaluate significant survival predictors with coefficient metrics that are easy to interpret. However, the CPH model relies on a manual selection of the most important features with potential omittance of important features. Therefore, the CPH model may not be able to handle complex and vast data with multidimensional features. Machine learning methods have shown since their application in econometrics and biosciences that it is especially useful for prediction problems (Jordan and Mitchell 2015). Using the *c*-index as a measure of performance, the random survival forest (RSF) model achieved the most accurate prediction with a *c*-index of 0.736. This value is generally considered in the setting of time-dependent censored data such as long-term survival as a good to strong prediction model. Moreover, we could show that the RSF algorithm was able to outperform the CPH model as well as all other machine learning models. In our setting, the RSF is robust which is proofed by 3-, 4- and 5-fold cross-validation with 5 repeats each (data not shown). We therefore believe that the RSF model is the most suitable machine learning algorithm to predict oncological outcome after curative resection of gastroesophageal adenocarcinoma.

The strength as well as the limitation of this study is the availability of detailed and high-resolution data for each patient. On the one hand, it was possible to establish an excellent prediction model under these circumstances. On the other hand, it might not be feasible in every setting to extract all presented data for each patient who is treated in a surgical hospital. To establish a more practical approach, we selected the 20 most important features identified by the RSF. By reducing the necessary input parameters from 117 to 20 and thus avoiding any tedious or unnecessary data management, it may be possible to make oncological predictions more practicable for the surgical oncologist. The resulting test statistic of this compact RSF model performs in a comparable strength and could be applied in the future. In both cases, extended or compact RSF, the test statistic outperformed the Cox proportional hazards model. It is also imaginable to establish an online application similar to the surgical risk calculator by the American College of Surgeons (Bilimoria et al. 2013) enabling clinicians to enter the 20 variables anonymously, since no identifying information is needed to handle the algorithm. The application can then generate an individual assessment based on the information that is present at the time of discharge. Given the anonymous information, it would also be possible to show specific survival curves according to the trained model and the risk group that the patient belongs to. This is statistically legitimate since the plotted survival curve represents the survival function for all patients with the same risk score range over the time span of 10 years.

The application of an individual and numeric risk score summarizes all available clinical and histopathological data that is predictive for long-term oncological outcome. Future clinical trials could rely on this risk score to find differences for overall survival which is the actual outcome parameter of primary interest. Thus, it is imaginable that subgroups could be treated with or without adjuvant therapy not according to single features such as TNM-status but based on the affiliation to the risk groups presented in this study. Likewise, high-risk patients with worst prognosis could be analyzed separately to eventually achieve improvements in surveillance and treatment. In this context, it is urgent to identify and characterize high-risk patients with unfavorable histopathological and postoperative parameters who still show a comparably good survival. To this date, it is not properly understood if the mere fact that the tumor was resected may nevertheless have an impact on overall survival for this specific patient collective and it is debatable if immunological reactions play a role. It is also necessary to implement a generally accepted risk score such as the presented machine learning model regardless of resectional status to offer a prognostic tool also for palliative situations.

This study is outstanding due to a systematic follow-up, high data quality and a thorough, state-of-the-art imputation with application of documented Python packages. In regard to most medical publications that often do not even deal with missingness at all, the overall data missingness of 1.3% is considered to be comparatively low. Moreover, we imputed the missing data via IterativeImputer from the scikit-learn package. IterativeImputer is a multivariate imputation method which estimates each missing feature from all other existing data points. Thus, it is possible to impute missing values by modeling each missing feature as a function of other features in a round-robin fashion. Even though data missingness is in general a limitation, this very low number of missing data points and our elaborate way of imputation both stand out in contrast to other works with simple data point deletion or imputation via means. All reported machine learning models are retrievable and reproducible via open source and have been cited accordingly. However, the clinical relevance of the reported RSF model as well as the other machine learning models needs to be clarified in prospective analyses with inevitably necessary external validation. Furthermore, the value differences between the several survival forest models are limited to some hundredths of the *c*-index and the discussion of these differences may be of limited clinical relevance. Nevertheless, we could show in a supervised learning setting that the algorithms were able to automatically select and extract predictors that are verifiably relevant for long-term prognosis.

## Conclusions

The results of our study demonstrate how machine learning algorithms can outperform usual prediction models for oncological outcome after resection of gastroesophageal tumors. Our results suggest that compared to other methods the random survival forest is most appropriate to predict long-term prognosis. We provide a clinical tool for clinicians which has to be validated based on further collectives in the future. In the future, interventional trials could be designed in a way that outcome parameters such as the presented risk score are evaluated to measure therapeutic interventions.

## Supplementary Information

Below is the link to the electronic supplementary material.

Supplementary file1 (DOCX 23 KB)

Supplementary file2 (DOCX 1673 KB)
