# Supplementary material for: Machine learning for optimized individual survival prediction in resectable upper gastrointestinal cancer
Source: J Cancer Res Clin Oncol. 2022 May 26;149(5):1691–702. doi: 10.1007/s00432-022-04063-5 (PMC10097798; doi:10.1007/s00432-022-04063-5)
Supplement: Supplementary file 1 — Supplementary file1 (DOCX 23 KB) [file 432_2022_4063_MOESM1_ESM.docx]

| *n = 1,360* | **Positive / Negative (Frequency)** | **Missingness** |
| --- | --- | --- |
| **Past medical history** | | |
| Severe diseases | 378 / 979 (27.86%) | 3 (0.2%) |
| Cardiovascular diseases | 620 / 738 (45.66%) | 2 (0.1%) |
| Pulmonary diseases | 164 / 1194 (12.08%) | 2 (0.1%) |
| Metabolic diseases | 286 / 1072 (21.06%) | 2 (0.1%) |
| Arterial hypertension | 536 / 823 (39.44%) | 1 (0.1%) |
| Congestive heart disease | 153 / 1206 (11.26%) | 1 (0.1%) |
| Asthma bronchiale | 46 / 1313 (3.38%) | 1 (0.1%) |
| COPD | 64 / 1295 (4.71%) | 1 (0.1%) |
| Renal insufficiency | 33 / 1326 (2.43%) | 1 (0.1%) |
| Hypothyreosis | 71 / 1287 (5.23%) | 2 (0.1%) |
| Hyperthyreosis | 23 / 1336 (1.69%) | 1 (0.1%) |
| Diabetes mellitus | 159 / 1200 (11.70%) | 1 (0.1%) |
| Peripheral artery disease | 81 / 1278 (5.96%) | 1 (0.1%) |
| **Neoadjuvant therapy** | | |
| Platin component | 613 / 725 (45.81%) | 22 (1.6%) |
| Taxane component | 435 / 898 (32.63%) | 27 (2.0%) |
| Epirubicin component | 195 / 1136 (14.65%) | 29 (2.1%) |
| Canceled neoadjuvant therapy | 77 / 1275 (5.70%) | 8 (0.6%) |
| **Postoperative complications** | | |
| Surgical complication | 418 / 940 (30.78%) | 2 (0.1%) |
| Major postoperative complication | 275 / 1085 (20.22%) | 0 (0.0%) |
| Pneumothorax | 43 / 1308 (3.18%) | 9 (0.7%) |
| Pleural effusion | 109 / 1242 (8.07%) | 9 (0.7%) |
| Anastomotic leakage | 161 / 1199 (11.84%) | 0 (0.0%) |
| Ischemia of enteral interposition | 15 / 1345 (1.10%) | 0 (0.0%) |
| Duodenal leakage | 15 / 1345 (1.10%) | 0 (0.0%) |
| Tracheoesophageal fistula | 4 / 1356 (0.29%) | 0 (0.0%) |
| Chylothorax | 8 / 1352 (0.59%) | 0 (0.0%) |
| Lesion of recurrent laryngeal nerve | 9 / 1349 (0.66%) | 2 (0.1%) |
| Bowel perforation | 14 / 1346 (1.03%) | 0 (0.0%) |
| Hemodynamical bleeding | 49 / 1311 (3.60%) | 0 (0.0%) |
| Pancreatic fistula | 22 / 1338 (1.62%) | 0 (0.0%) |
| Pancreatitis | 16 / 1343 (1.18%) | 1 (0.1%) |
| Peritonitis | 29 / 1331 (2.13%) | 0 (0.0%) |
| Intraabdominal abscess | 83 / 1277 (6.10%) | 0 (0.0%) |
| Dehiscence of abdominal fascia | 18 / 1340 (1.33%) | 2 (0.1%) |
| Cardiac complications | 152 / 1207 (11.18%) | 1 (0.1%) |
| Pulmonary complications | 287 / 1073 (21.10%) | 0 (0.0%) |
| Sepsis | 87 / 1267 (6.43%) | 6 (0.4%) |
| **Supplementary Table 1:** Additional information regarding prevalence and data missingness for the input categories past medical history, neoadjuvant therapy and postoperative complications. COPD = chronic obstructive pulmonary disease. | | |

| **Model** | **Hyperparameter** | **Value** |
| --- | --- | --- |
| Standard Cox Proportional Hazards | Initialization method  Maximum Iteration  Learning rate  L2 regularization parameter  Tolerance | zeros  50  1e-04  1e-04  1e-03 |
| Non-Linear Cox Proportional Hazards (DeepSurv) | Initialization method  Number of epochs  Learning rate  L2 regularization parameter  Optimizer | glorot uniform  500  1e-03  1e-04  adam |
| Linear Multi-Task Regression | Initialization method  Number of epochs  Learning rate  L2 regularization parameter  Secondary L2 parameter (smooth)  Optimizer | glorot uniform  1500  1e-03  1e-05  1e-05  adam |
| Neural Multi-Task Regression | Activation  Number of units  Initialization method  Number of epochs  Learning rate  Optimizer | ReLu  150  glorot uniform  1000  1e-04  adam |
| Gompertz (Parametric Model) | Initialization method  Number of epochs  Learning rate  L2 regularization parameter  Optimizer | glorot uniform  500  1e-03  1e-05  adam |
| Conditional Survival Forest | Number of trees  Alpha  Importance mode  Maximum features  Minimum node size  Minimum splitting quantile | 200  0.07  normalized permutation  all  15  0.1 |
| Extra Survival Trees | Number of trees  Importance mode  Maximum features  Minimum node size  Number of random splits | 200  impurity corrected  all  8  80 |
| Random Survival Forest | Number of trees  Importance mode  Maximum features  Minimum node size | 200  normalized permutation  all  5 |
| **Supplementary Table 2:** Hyperparameter selection after optimization via Halving Grid Search (scikit-learn) for all tested machine learning algorithms. | | |
