# Supplementary material for: Machine learning for optimized individual survival prediction in resectable upper gastrointestinal cancer
Source: J Cancer Res Clin Oncol. 2022 May 26;149(5):1691–702. doi: 10.1007/s00432-022-04063-5 (PMC10097798; doi:10.1007/s00432-022-04063-5)
Supplement: Supplementary file 2 — Supplementary file2 (DOCX 1673 KB) [file 432_2022_4063_MOESM2_ESM.docx]

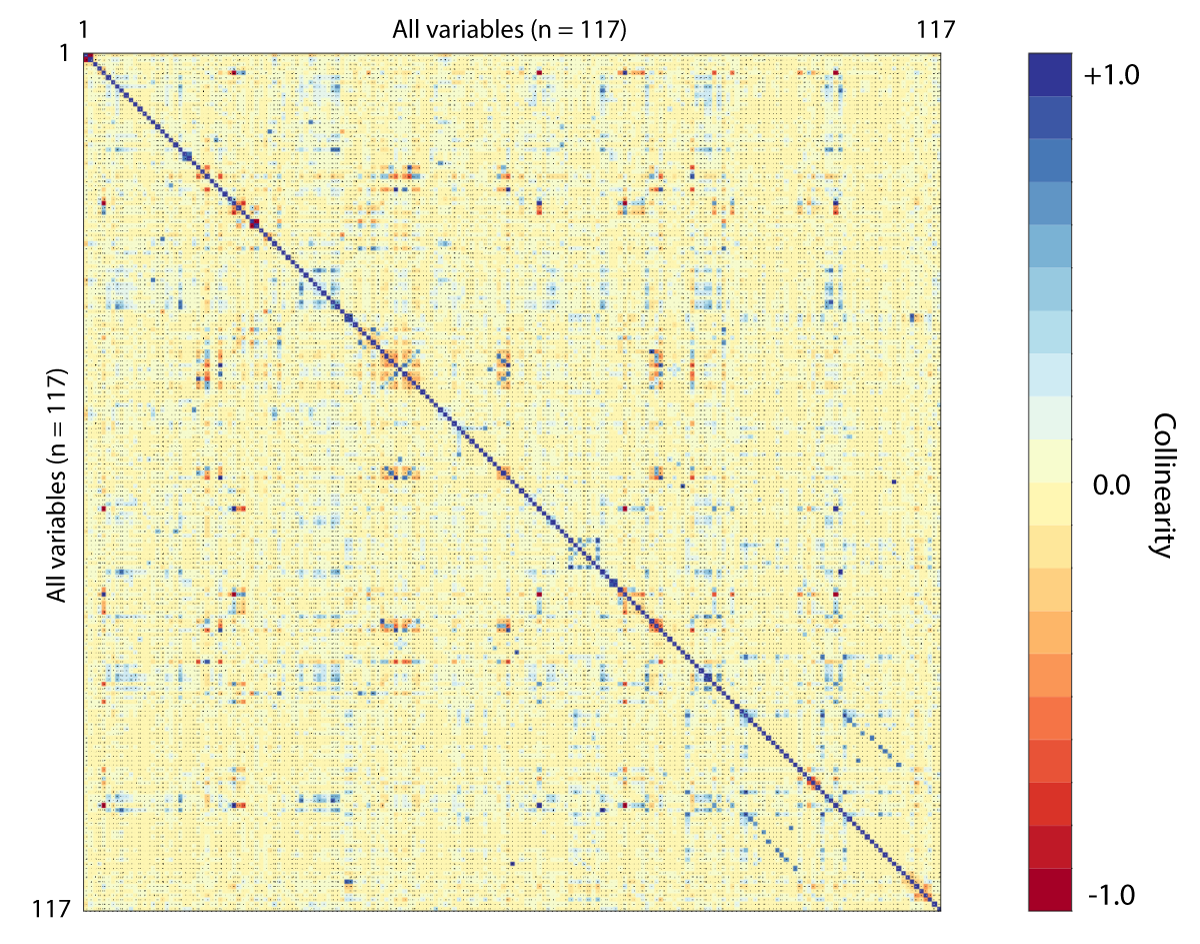


**Supplementary Figure 1:** Heatmap of all variables (117 x 117) depicting collinearity of all variables with isolated predictors which were excluded from further analysis.


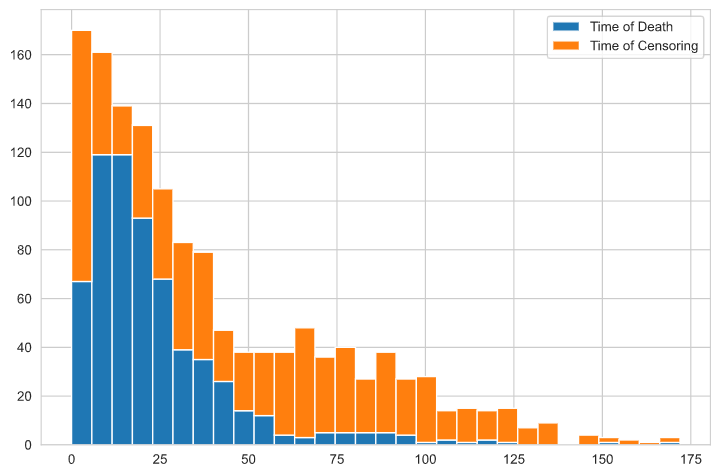


**Supplementary Figure 2:** Bar graph of censored versus documented data from systemic follow-up.


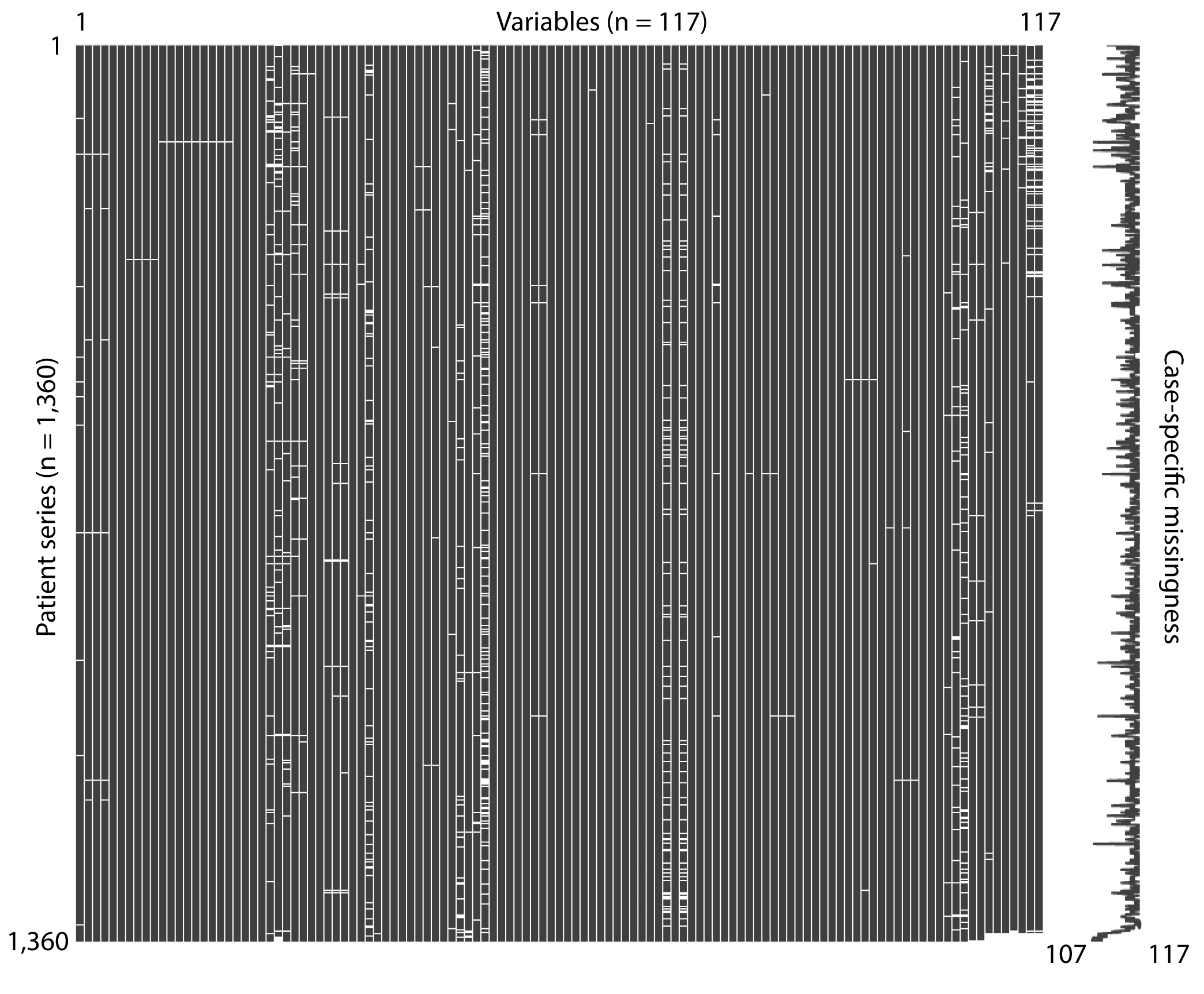


**Supplementary Figure 3:** Missingness matrix of all 117 variables (plotted on the x-axis) and a total of 1,360 cases (plotted on the y-axis) demonstrating a total missingness of 1.3%. The right-hand diagram shows the individual missingness with a maximum missingness in 10 variables for one patient resulting in a data availability in 107 variables.


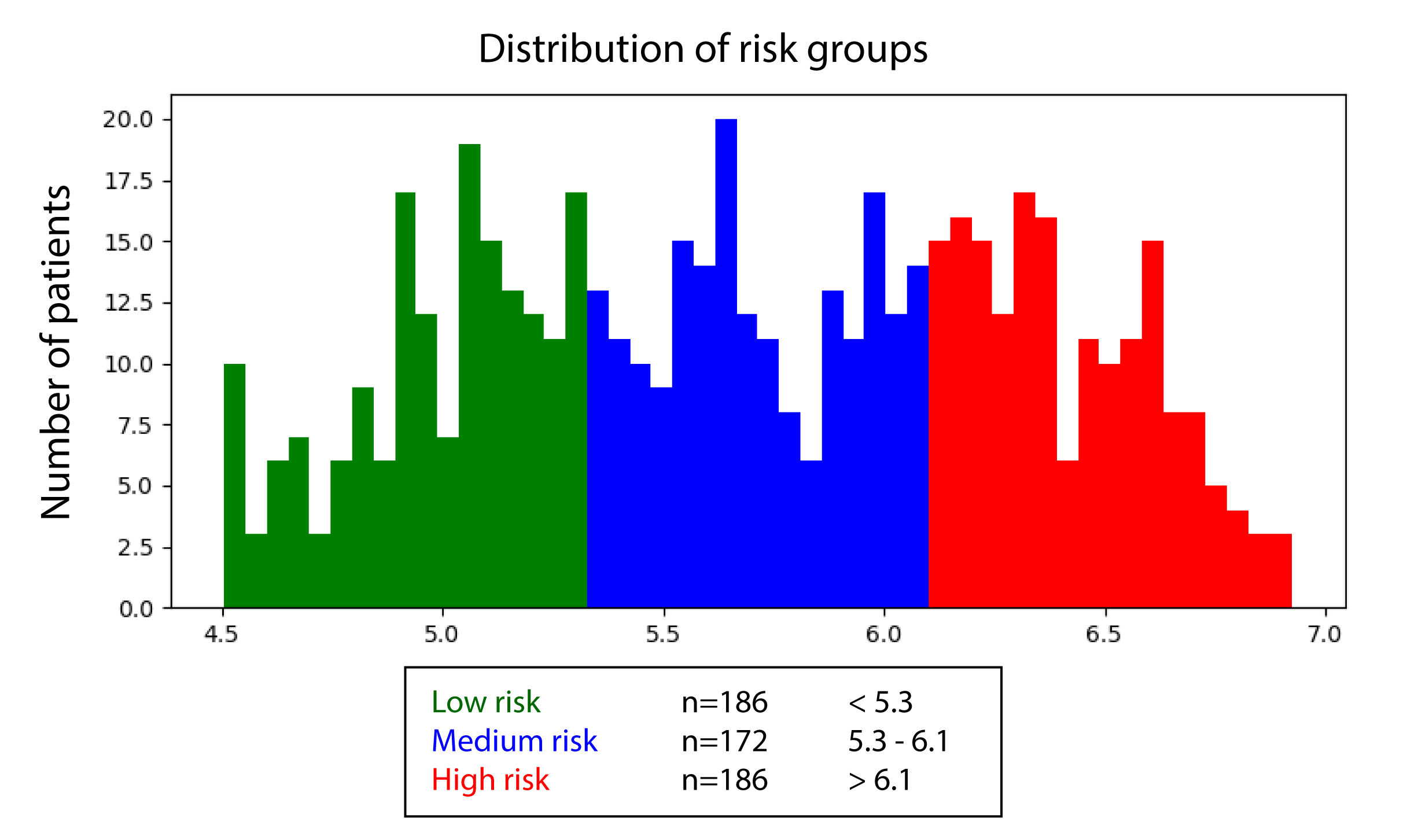


**Supplementary Figure 4:** Risk score distribution of the compact RSF model based on the 20 most important variables.


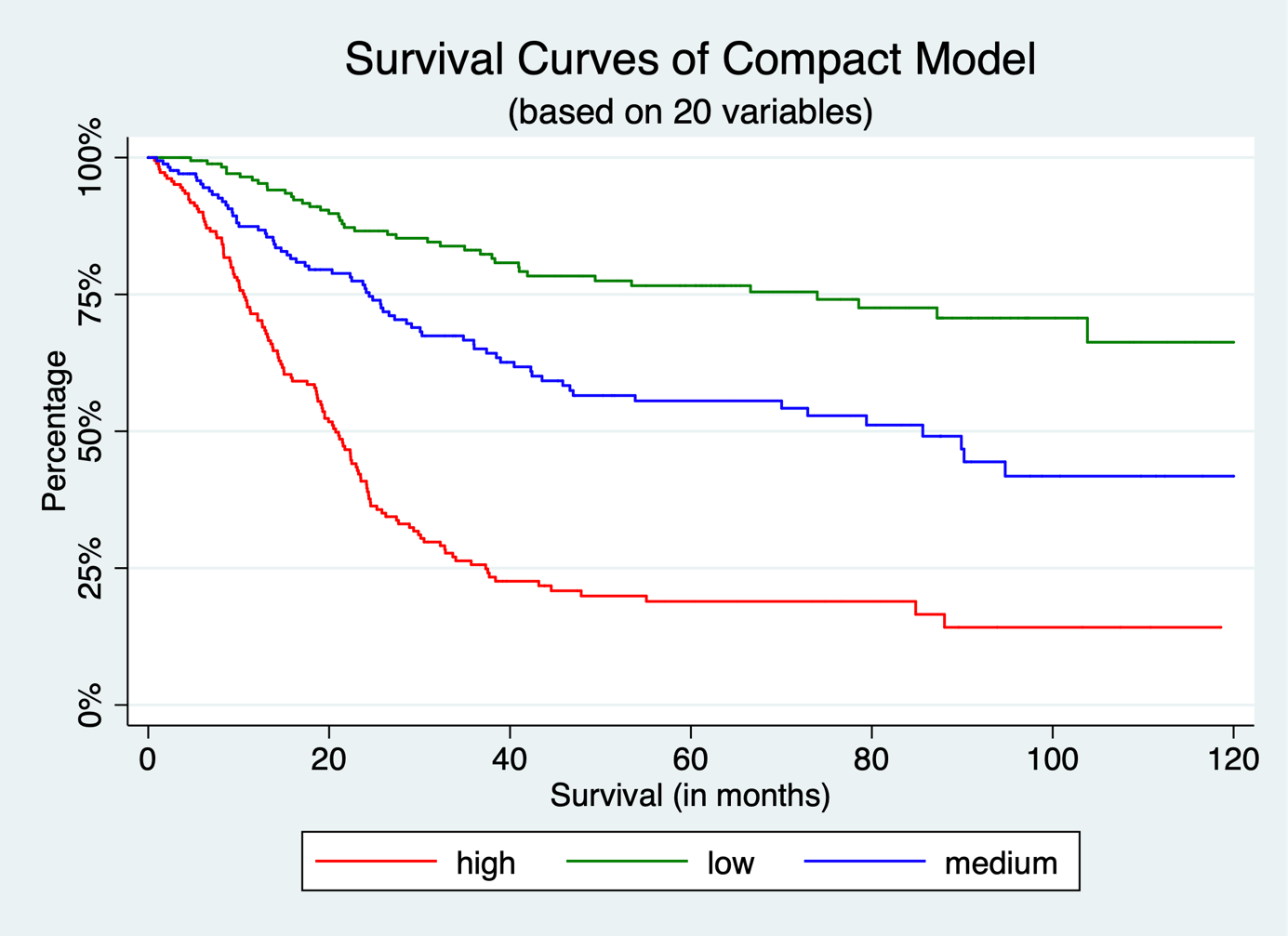


**Supplementary Figure 5:** Kaplan Meier plot of the compact RSF model and its three different risk groups.
